# Mechanism Underlying Light Intensity-Induced Melanin Synthesis of *Auricularia heimuer* Revealed by Transcriptome Analysis

**DOI:** 10.3390/cells12010056

**Published:** 2022-12-23

**Authors:** Zhiheng Qiu, Yanliang Gao, Shuang Wang, Jun Wang, Xinyi Wang, Nuo Cai, Jiazhi Zhao, Tingshu Li, Hongpeng Li, Tianlai Li, Lili Shu

**Affiliations:** 1 College of Horticulture, Shenyang Agricultural University, Shenyang 110866, China; 2 Key Laboratory of Protected Horticulture of Education Ministry and Liaoning Province, Shenyang 110866, China

**Keywords:** *Auricularia heimuer*, color, light intensity, transcriptional profiles, melanin synthesis

## Abstract

*Auricularia heimuer* is a traditional edible and medicinal mushroom, which is widely used in biochemical research and is regarded as a good dietary supplement. The color of the ear-like fruiting body is an important indicator of its commercial quality. However, the mechanism by which light intensity influences the melanin synthesis of *A. heimuer* remains unclear. Here, we show that fruiting body color is significantly affected by light intensity. Transcriptional profiles of the fruiting bodies of *A. heimuer* grown in different light intensities were further analyzed. More differentially expressed genes (DEGs) were identified with a greater light intensity difference. A total of 1388 DEGs were identified from six comparisons, including 503 up-regulated genes and 885 down-regulated genes. The up-regulated genes were mainly associated with light sensing via photoreceptors, signal transduction via the mitogen-activated protein kinase (MAPK) signaling pathway, and melanin synthesis via the tyrosine metabolic pathway. Therefore, the genes involved in these processes may participate in regulating melanin synthesis under high light intensity. This insight into the transcriptional regulation of *A. heimuer* to light intensity should help to further comprehensively elucidate the underlying mechanism of light-induced melanin synthesis.

## 1. Introduction

*Auricularia heimuer*, commonly known as tree-ear, is one of the most widely cultivated and consumed edible mushrooms in Asia [1], with the second largest production among all edible mushrooms worldwide. The fruiting body of *A. heimuer* has an ear-like structure and is rich in gelatin along with a variety of bioactive substances, including polysaccharides, polyphenols, vitamins, and melanin [2]. Cap color is an important commercial characteristic for *A. heimuer*, with consumers preferring mushrooms with a blacker cap. Therefore, cultivating *A. heimuer* with black caps can improve growers’ profits; however, the fruiting body of *A. heimuer* shows a significant color difference under different light-intensity cultivation conditions.

Color is one of the most important sensory attributes of all dietary foods. Pigment is the decisive factor in color appearance [3], and melanin is one of the main pigments affecting biological color, which is a common pigment detected in fungi. For many fungi, melanin can enhance their adaptability to adverse environmental stresses, such as oxidizing agents, ultraviolet (UV) light, and ionizing radiation [4]. In addition, the melanin produced by many fungi has a variety of functional properties and biological activities, such as a hepatoprotective effect, a hypolipidemic effect, and an anti-cancer effect [5].

Melanin is a complex heterogeneous polymer consisting of phenolic and/or indolic acid monomers, which is located on the fungal cell wall and is tightly cross-linked to cell wall polysaccharides [6]. There are two main melanin biosynthesis pathways in fungi: the 1,8-dihydroxynaphthalene (DHN) and _L_-3,4-dihydroxyphenylalanine (_L_-DOPA) pathways [7]. A previous study demonstrated that the main components of melanin in *Auricularia auricula* are eumelanin, pheomelanin, and DHN melanin [8]. The fungal melanin biosynthesis pathway involves multiple redox, dehydration, and polymerization reactions with the participation of multiple enzymes, each of which is encoded by several different genes. Many previous studies have found that environmental stimuli play important roles in the melanin synthesis of *A. heimuer*, including light, high temperature, and radiation [9,10].

Light is one of the essential environmental factors required for the growth and development of higher fungi, playing an important role in regulating their physiological cycle, morphogenesis, and metabolite synthesis [11]. Fungi can sense variations in light wavelengths and intensity using multiple photoreceptors, leading to intracellular transcriptional responses to regulate the expression of genes that can enable adaption to environmental changes or stresses [12]. Indeed, most light-induced responses ultimately depend on gene regulation. Light signaling can be transmitted to cells through photoreceptors to cause gene expression or suppression in filamentous fungi, leading to changes in metabolic and morphogenetic pathways [13]. For instance, low-intensity, coherent blue light was reported to stimulate the melanin synthesis of the chaga fungus *Inonotus obliquus* [14]. Previous studies on the melanin of *A. heimuer* have mainly focused on the effects of various nutrients on melanin synthesis, melanin extraction, and biological activities [15,16]. However, studies on the gene regulation of melanin synthesis in *A. heimuer*, especially the light intensity-dependent regulation mechanism, remain limited. RNA-sequencing (RNA-seq) is a common and effective method for determining the transcriptional expression profile of mushrooms under various light treatments [17]. For instance, Yoo et al. [18] successfully identified candidate genes involved in the light-induced formation of the mycelial brown film using comparative transcriptome analysis.

Therefore, the aim of this study was to gain insight into the light intensity-dependent regulation of melanin synthesis in *A. heimuer* at the molecular level. Illumina sequencing technology was used to detect the differentially expressed genes (DEGs) of *A. heimuer* cultivated under different light intensities and the impact on melanin content and color. To our knowledge, this is the first study to directly explore the effect of light intensity on melanin synthesis in *A. heimuer*. The obtained light intensity-dependent transcriptional expression profile of *A. heimuer* will thus establish a critical basis for elucidating the regulatory mechanism of melanin synthesis.

## 2. Materials and Methods

### 2.1. Strain, Culture Conditions and Sample Preparation

The *A. heimuer* strain (CCMJ 1252) was obtained from the Culture Collection Center of Mycology of Jilin Agriculture University. For the pure culture of *A. heimuer*, a 5-mm-diameter punch from solid medium was inoculated onto the center of potato dextrose agar (Difco, Becton-Dickinson Co., Sparks, MD, USA) and incubated at 25 °C. To grow the mycelia, five mycelia discs (7 mm) were inoculated into a sawdust medium (82% sawdust, 12% bran, 2% soybean meal, 2% corn meal, 1% lime, 1% gypsum) in a polyethylene bag (17 × 15 cm) and incubated at 25 °C. Each bag contained 1.5 kg of medium with 60% humidity. Sunlight-like light-emitting diode light strips were used as the light source. The samples were divided into four treatment groups according to light intensity (B1: 10 μmol·m^−2^·s^−1^; B2: 50 μmol·m^−2^·s^−1^; B3: 250 μmol·m^−2^·s^−1^; B4: 500 μmol·m^−2^·s^−1^). To collect the fruiting body of *A. heimuer*, the management of the whole cultivation cycle was applied according to a previously reported method [19]. The individual fruiting bodies were analyzed in triplicate for each light intensity treatment, frozen immediately in liquid nitrogen, and then stored at –80 °C until RNA extraction. A portable colorimeter (Dongguan Zhongsheng Instrument Co., Ltd., Dongguan, Guangdong, China) was used to detect the color differences between the fruiting bodies collected from different light intensity treatments.

### 2.2. Isolation of Melanin from the Fruiting Body of A. heimuer

*A. heimuer* melanin was extracted and purified from fruiting bodies with different colors collected from different light intensity treatments according to a previously reported method with minor modifications [20]. Briefly, the fruiting bodies of *A. heimuer* were crushed by a homogenizer. The obtained fruiting body power (20 g) was then mixed with 1.5 M NaOH at a volume ratio of 1:30 (power:NaOH solution). The suspension was treated with a sonicator (Thermo Fisher Scientific, Carlsbad, CA, USA) at 160 W for 90 min and left to stand overnight at room temperature. After centrifuging at 16,000 rpm for 15 min, the supernatant was transferred to a beaker and the pH was adjusted to 1.5 with 7 M HCl. The supernatant was left to stand at room temperature for 3 h to fully precipitate. The precipitate was then collected by centrifugation at 16,000 rpm for 15 min and washed repeatedly with deionized water until the pH reached a neutral level.

To purify the melanin, the crude melanin was suspended in 7 M HCl and maintained at 100 °C for 2 h. The precipitate was then collected by centrifugation at 16,000 rpm for 15 min and washed with deionized water three times. Subsequently, the precipitate was washed with absolute ethanol, chloroform, and ethyl acetate, and finally, the ethyl acetate in the precipitate was blown dry at room temperature. The dried precipitate was dissolved in 2 M NaOH and magnetically stirred overnight. The supernatant was collected at 16,000 rpm for 15 min, and the pH was adjusted to 1.5 with 7 M HCl for precipitation. The precipitate was collected by centrifugation at 16,000 rpm for 15 min and washed with deionized water until achieving a neutral pH. Finally, the pure melanin was collected and lyophilized (Christ ALPHA 1-2 LD plus, Martin Christ Gefriertrocknungsanlagen GmbH, Osterode am Harz, Germany).

### 2.3. RNA Isolation

Total RNA was extracted from the fruiting body using the RNeasy Plant Mini Kit (Qiagen, Inc., Valencia, CA, USA) according to the manufacturer’s protocol. The NanoDrop 3300 fluorospectrometer (Thermo Fisher Scientific, Waltham, MA, USA) and Agilent 2100 Bioanalyzer were used to confirm the concentrations and quality of RNA samples. The integrity of the RNA extracts was confirmed by the Agilent 2100 RNA Nano 6000 assay kit (Agilent Technologies, Santa Clara, CA, USA).

### 2.4. cDNA Library Construction and Illumina Sequencing

A total of 1 μg of RNA was used for library preparation. Transcriptome sequencing libraries were constructed with the VAHTS^®^ Universal V8 RNA-seq Library Prep Kit for Illumina (Illumina, San Diego, CA, USA). Briefly, the poly(A) mRNA was enriched using oligo(dT) beads. mRNA fragmentation was performed using divalent cations under elevated temperature in an Illumina proprietary fragmentation buffer. Random primers were performed for priming in the kit. In this way, first and second-strand cDNA were synthesized. After purifying double-stranded cDNA, both ends were repaired, and a dA-tailing was added in one reaction, followed by adding adaptors to both ends. Then, DNA Clean BeadsSize was used for selection of adaptor-ligated DNA. Each sample was then amplified by PCR using the P5 and P7 primers and the PCR products were validated. Library concentrations were quantified using a Qubit 2.0 fluorometer (Life Technologies, Carlsbad, CA, USA). Then, twelve cDNA libraries with different indices were multiplexed and loaded on an Illumina NOVAseq 6000 platform for sequencing. Three independent sequencing libraries were prepared for each light intensity treatment. The raw sequence data were submitted to the National Center for Biotechnology Information (NABI) Sequence Read Archive (SRA) under accession number SUB12166970.

### 2.5. RNA-Seq Data Analysis

The raw sequence data were provided in fastq format. High-quality clean data were generated by removing technical sequences (adapters, PCR primers, or fragments thereof; quality of bases lower than 20) with Cutadapt (V1.9.1, https://pypi.org/project/cutadaptor/, accessed on 11 November 2022) [21]. The trimmed, clean data were aligned to the *A. heimuer* reference genome (SAMN26443385) via the Hisat2 software (V2.0.1, http://daehwankimlab.github.io/hisat2/main/, accessed on 11 November 2022). Htseq (V0.6.1, https://pypi.org/project/HTSeq/, accessed on 11 November 2022) was used to estimate gene and isoform expression levels from the pair-end clean data. The DESeq2 Bioconductor package (V1.36.0, https://bioconductor.org/packages/release/bioc/html/DESeq2.html, accessed on 11 November 2022) was used to identify DEGs based on the negative binomial distribution [22]. The abundance of sample mRNA was quantified using fragments per kilobase of exon per million mapper reads (FPKM) of each gene. The resulting *p* values for comparisons of expression levels were adjusted with the Benjamini and Hochberg method to control the false discovery rate (corrected *p* < 0.05) [23]. To identify genes responding to different light intensities, a gene with an adjusted *p* value < 0.05 and an absolute value of log2 fold change > 1 was considered to be a significant DEG.

### 2.6. Functional Annotation and Pathway Analysis of DEGs

The assembly of all the reads and gene function annotation were all performed based on a previous study [24]. GO and KEGG annotations were used for enrichment analyses of the identified DEGs. GOSeq (V1.34.1) was used to identify GO terms with an annotated list of enriched genes according to a significant adjusted *p* value. GO terms with a corrected *p* value < 0.05 were considered to be significantly enriched by DEGs. The ClusterProfile R package was used for the statistical enrichment of DEGs in KEGG pathways.

### 2.7. Valiation of RNA-Seq Results by Quantitative Real-Time PCR (qRT-PCR)

qRT-PCR assays were performed to validate the quality of the RNA-seq results. According to the differential expression profiles of genes between different light intensity samples, qRT-PCR on a Bio-Rad CFX96 System (Bio-Rad Laboratories, Inc., Hercules, CA, USA) was used to validate eight randomly selected DEGs, including genes related to light, genes in signal pathways related to light, and genes involved in melanin synthesis pathways. Total RNA extraction was performed as described above. Each sample was analyzed in triplicate. The *TransStart*^®^ Top Green qPCR SuperMix (+Dye II) (TransGen Biotech Co., LTD, Beijing, China) was used according to the manufacturer’s protocol. The primers, which are listed in Table S1, were designed with the Primer Premier 6 software. The *GADPH* gene of *A. heimuer* was used as the internal control gene. The relative gene expression level was calculated using the 2^−ΔΔCt^ method. 

### 2.8. Statistical Analysis

All statistical analyses were analyzed using SPSS 20.0 (SPSS Inc., Chicago, IL, USA). Data are presented as the means ± standard deviation (SD) from at least three biological replicates. Statistically significant differences were determined by analysis of variance (ANOVA) and Duncan’s multiple range tests (*p* ≤ 0.05).

## 3. Results

### 3.1. High Light Intensity Induced Melanin Synthesis in A. heimuer

**Figure 1:**
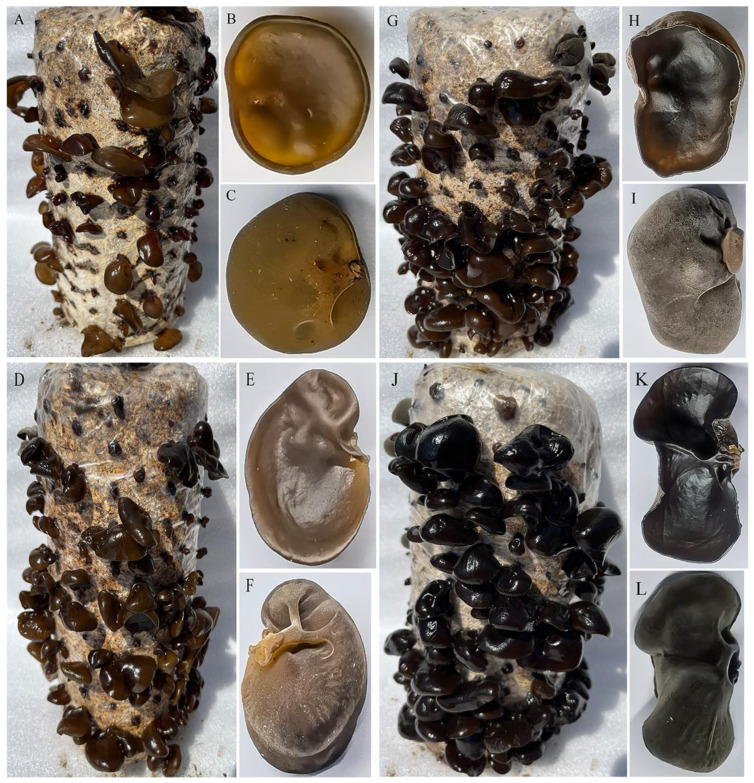
Color features of the fruiting body collected from different light intensity treatments. (**A–C**) Color of the fruiting bodies grown under low light intensity (B1: 10 μmol·m^−2^·s^−1^). (**D–F**) Color of the fruiting bodies grown under the light intensity of 50 μmol·m^−2^·s^−1^ (B2). (**G–I**) The color of the fruiting bodies grew under the light intensity of 250 μmol·m^−2^·s^−1^(B3). (**J–L**) The color of the fruiting bodies grew under the light intensity of 500 μmol·m^−2^·s^−1^ (B4).

The fruiting body color of *A. heimuer* grown under varying light intensities (B1, 10 μmol·m^−2^·s^−1^; B2, 50 μmol·m^−2^·s^−1^; B3, 250 μmol·m^−2^·s^−1^; B4, 500 μmol·m^−2^·s^−1^) differed significantly. With the increase in light intensity, the color of the fruiting body showed a trend toward becoming darker and darker (Figure 1). Compared with fruiting bodies growing under low light intensity, those growing under high light intensity showed a more obvious black color (Figure 1J–L). Under the B1 treatment (low light intensity), the L* value of the fruiting bodies was the largest among the four treatments, indicating the lowest degree of black. In contrast, the fruiting bodies growing under the B4 treatment (high light intensity) showed the lowest L* values (Table 1). 

**Figure 2:**
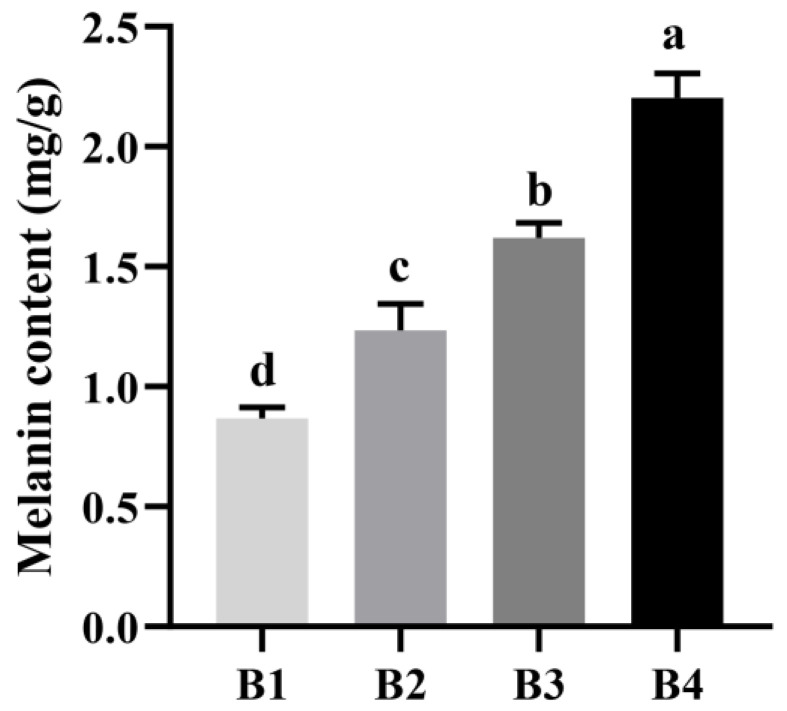
Melanin content in fruiting bodies grown at different light intensities.

This color variation was reflected by the measurement of melanin content in the fruiting bodies collected from the four light intensity treatment groups. As shown in Figure 2, the highest melanin content was detected in the B4 treatment sample, and the lowest was found in the B1 treatment sample. Thus, with the increase in light intensity, the melanin content in the fruiting body also increased, further suggesting that melanin content is correlated with sensory and color space values. Taken together, these results confirmed that an increase in light intensity will induce the synthesis of melanin and deepen the color of the fruiting bodies of *A. heimuer*.

### 3.2. Overview of the Transcriptomic Response to Different Light Intensities

A total of 12 complementary DNA (cDNA) libraries were constructed, and 600 million raw reads with 90 GB of raw data were obtained (Table S2). After excluding low-quality reads, a total of 523 million clean reads were generated (Table S3). The mapping rate of high-quality reads to the reference genome exceeded 80%, and the Q30 values of all cDNA libraries exceeded 90%. The boxplot of the fragments per kilobase of exon per million mapped fragments (FPKM) distribution of the different light intensity treatments showed a similar trend and concentrated range of values (Figure S1). These results confirmed that the transcriptomic data obtained were reasonable and suitable for subsequent analysis. Accordingly, the FPKM value was used to normalize the gene expression levels, and a total of 14,152 known genes were expressed in the B1, B2, B3, and B4 fruiting body libraries.

### 3.3. Identification of DEGs

**Figure 3:**
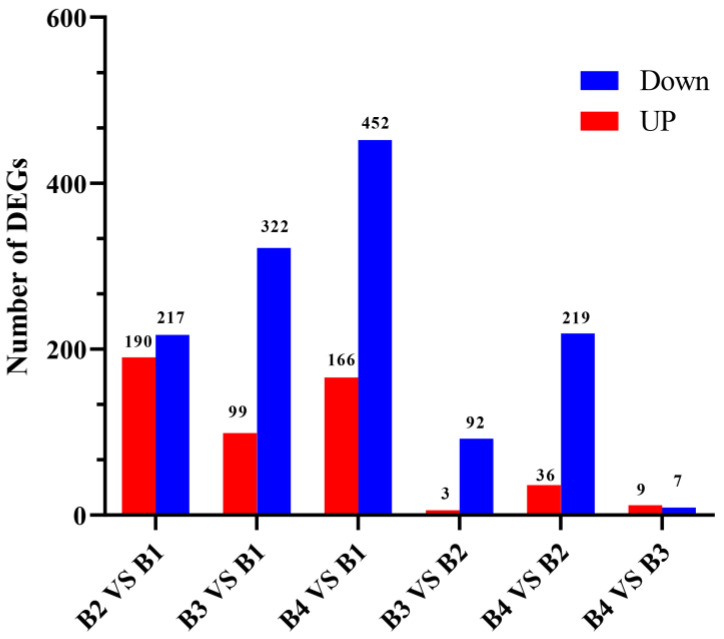
Number of DEGS between in different comparisons.

**Figure 4:**
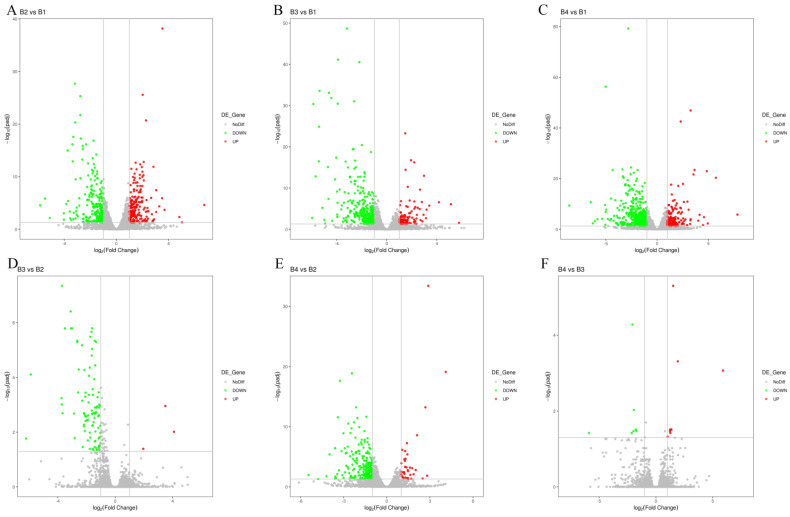
Overview of DEGs analyses for different light intensity treatments. (**A**) Volcano plot of significant DEGs between B2 vs. B1. (**B**) Volcano plot of significant DEGs between B3 vs. B1. (**C**) Volcano plot of significant DEGs between B4 vs. B1. (**D**) Volcano plot of significant DEGs between B3 vs. B2. (**E**) Volcano plot of significant DEGs between B4 vs. B2. (**F**) Volcano plot of significant DEGs between B4 vs. B3.

To identify the differential transcriptional profiles according to the different light intensity treatments (B1, B2, B3, and B4), the normalized read counts (FPKM values) were statistically compared, and DEGs were identified and annotated in six comparisons (B2 vs. B1, B3 vs. B1, B4 vs. B1, B3 vs. B2, B4 vs. B2, and B4 vs. B3) (Figure 3). Compared with the B1 treatment, 407 DEGs were identified in the B2 treatment (190 up-regulated and 217 down-regulated) (Table S4), 421 DEGs were identified in the B3 treatment (99 up-regulated and 322 down-regulated) (Table S5), and 618 DEGs were identified in the B4 treatment (166 up-regulated and 452 down-regulated) (Table S6). Compared with the B2 treatment, only 95 DEGs were identified in the B3 treatment (three up-regulated and 92 down-regulated), whereas 255 DEGs were identified in the B4 treatment (36 up-regulated and 219 down-regulated). In the comparison of B4 vs. B3, 16 DEGs were identified (9 up-regulated and 7 down-regulated). The Venn diagram in Figure S2 shows the overlap of significant DEGs in each of the three combinations (B2 vs. B1, B3 vs. B1, and B4 vs. B1). The common or unique significant DEGs of the three comparisons were screened (Table S7). As the light intensity difference increased, more DEGs were identified, with the highest number of DEGs identified in the B4 vs. B1 comparison (Figure 4), representing the largest light intensity difference. The heat map and sample cluster diagram were constructed based on the DEGs identified in different samples (Figure S3), which confirmed that light intensity could affect the expression of several genes. Taken together, these results suggest that these significant DEGs may play important roles in the melanin synthesis of *A. heimuer*.

### 3.4. Functional Annotation of DEGs

**Figure 5:**
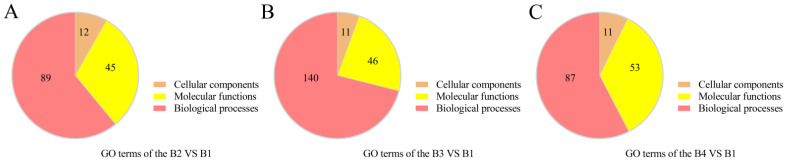
Overview of GO terms associated with the DEGs of the different comparisons. (**A**) GO terms enriched in the B2 vs. B1 comparison. (**B**) GO terms enriched in the B3 vs. B1 comparison. (**C**) GO terms enriched in the B4 vs. B1 comparison.

**Figure 6:**
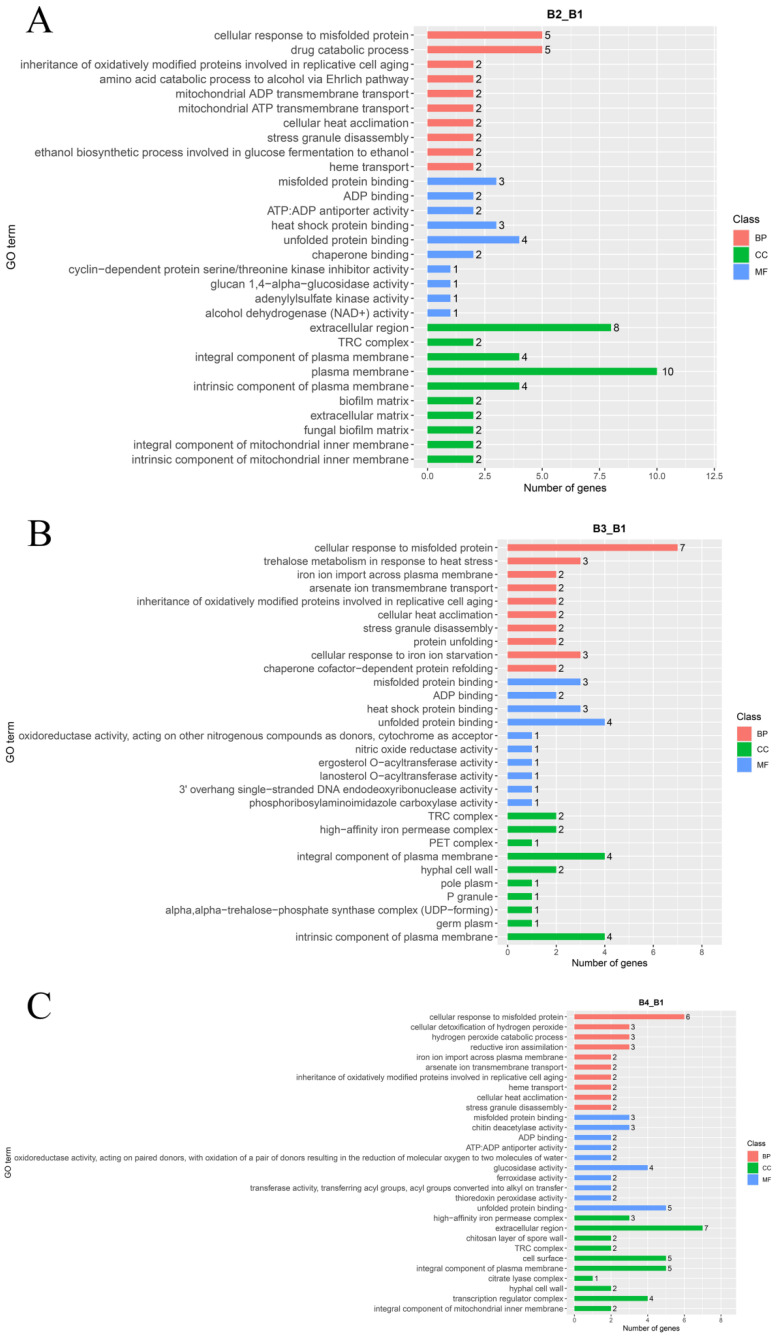
GO classification maps of DEGs of the (**A**) B2 vs. B1 (B2_B1), (**B**) B3 vs. B1 (B3_B1), and (**C**) B4 vs. B1 (B4_B1) comparisons.

For a better understanding of the functions of DEGs involved in the light intensity-induced melanin synthesis of *A. heimuer*, gene ontology (GO) enrichment analysis and Kyoto Encyclopedia of Genes and Genomes (KEGG) annotation were performed. GO enrichment analysis showed that the 407 DEGs in the B2 vs. B1 comparison were enriched in 146 terms, including 89 biological process (BP), 12 cellular component (CC), and 45 molecular function (MF) terms (Figure 5A). The top 30 enriched GO terms were mainly associated with the plasma membrane (GO:0005886, 10 genes), fungal biofilm matrix (GO: 0062040, two genes), unfolded protein binding (GO: 0051082, 4 genes), heat shock protein binding (GO: 0031072, three genes), cellular response to misfolded protein (GO: 0071218, five genes), and heme transport (GO: 0015886, two genes) (Figure 6A, Table S8).

The DEGs identified in the comparison of B3 vs. B1 were enriched in 197 terms, including 140 BP, 11 CC, and 46 MF terms (Figure 5B). The top 30 enriched terms were mostly associated with cellular responses to misfolded protein (GO: 0071218, seven genes), response to abiotic stimulus (GO: 0009628, 12 genes), oxidation-reduction process (GO: 0055114, 11 genes), multi-organism process (GO: 0051704, 10 genes), response to reactive oxygen species (GO: 0000302, five genes), response to osmotic stress (GO: 0006970, six genes), and oxidoreductase activity (GO: 0016491, nine genes) (Figure 6B, Table S9).

The DEGs identified in the comparison of B4 vs. B1 were enriched in a total of 151 terms, including 87 BP, 11 CC, and 53 MF terms (Figure 5C, Table S10). The top 30 enriched terms were mainly associated with cellular response to oxidation-reduction process (GO: 0055114, 20 genes), positive regulation of transcription by RNA polymerase II (GO: 0045944, eight genes), response to abiotic stimulus (GO: 0009628, nine genes), small molecule biosynthetic process (GO: 0044283, 11 genes), response to osmotic stress (GO: 0006970, four genes), and cellular response to chemical stress (GO: 0062197, 12 genes). 

**Figure 7:**
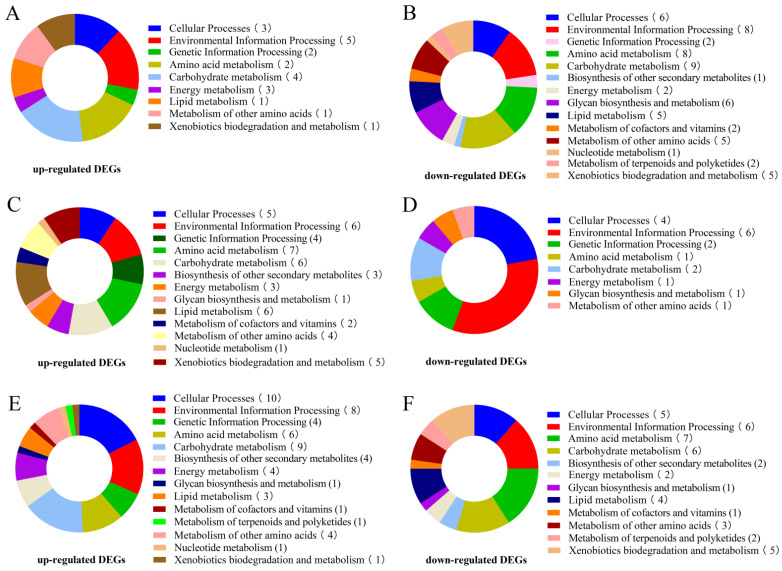
Enrichment of DEGs in KEGG pathways. (**A**) Functional KEGG categories enriched by up-regulated DEGs in B2 vs. B1. (**B**) Functional KEGG categories enriched by down-regulated DEGs in B2 vs. B1. (**C**) Functional KEGG categories enriched by up-regulated DEGs in B3 vs. B1. (**D**) Functional KEGG categories enriched by down-regulated DEGs in B3 vs. B1. (**E**) Functional KEGG categories enriched by up-regulated DEGs in B4 vs. B1. (**F**) Functional KEGG categories enriched by up-regulated DEGs in B4 vs. B1.

**Figure 8:**
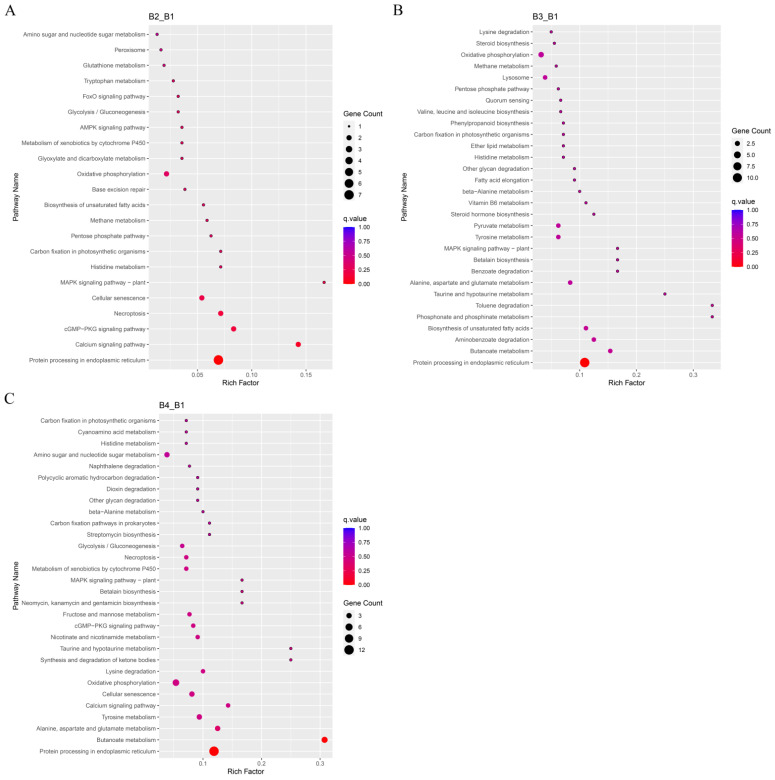
KEGG functional annotation map of significantly up-regulated differentially expressed genes (DEGs). (**A**) Enriched KEGG pathways in DEGs between B2 and B1 (B2_B1). (**B**) Enriched KEGG pathways in DEGs between B3 and B1 (B3_B1). (**C**) Enriched KEGG pathways in DEGs between B4 and B1 (B4_B1).

The KEGG pathways with the highest number of significant DEGs in *A. heimuer* treated with different light intensities were analyzed. The B1 sample was used as the control group to identify significant DEGs and perform KEGG enrichment analysis. The results of the KEGG pathway enrichment analysis between different comparisons (B2 vs. B1, B3 vs. B1, and B4 vs. B1) are displayed in Figure S4 and Tables S11–S13. The DEGs influenced by high light intensity were mainly classified into cellular processes, environmental information processing, genetic information processing, amino acid metabolism, and energy metabolism (Figure 7). The top 30 KEGG pathways enriched in the significantly up-regulated DEGs included tyrosine metabolism (ko00350), metabolism of xenobiotics by cytochrome P450 (ko00980), peroxisome (ko04146), mitogen-activated protein kinase (MAPK) signaling pathway (ko04016), protein processing in the endoplasmic reticulum (ko04141), oxidative phosphorylation (ko00190), and calcium signaling pathway (ko04020) (Figure 8, Tables S14–S16). Some significantly up-regulated DEGs were commonly enriched in some pathways in the three comparison groups (B2 vs. B1, B3 vs. B1, and B4 vs. B1), including pentose phosphate pathways, oxidative phosphorylation, amino sugar and nucleotide sugar metabolism, metabolism of xenobiotics by cytochrome P450, the MAPK signaling pathway, and protein processing in the endoplasmic reticulum, suggesting that these pathways may participate in melanin synthesis with the increase in light intensity. Moreover, up-regulated DEGs in tyrosine metabolism pathways were significantly enriched with the increase of light intensity (B3 vs. B1 and B4 vs. B1) (Figure 8B,C).

### 3.5. Genes Related to Light

**Figure 9:**
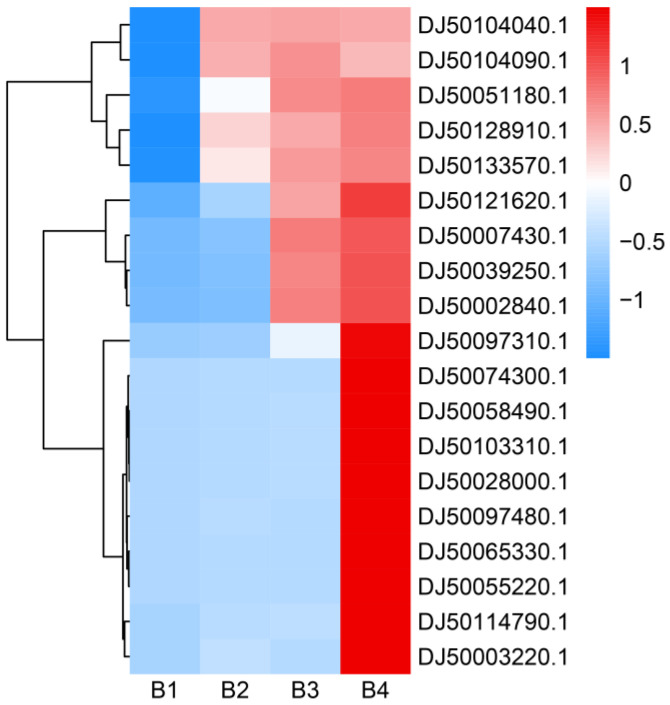
Heatmap analysis of gene expression levels under different light intensities. The right side of the heatmap indicates the gene ID. The FPKM values were transformed to Z-score values. The higher the gene expression level, the higher the Z-score value. The heatmap was drawn and colored in R language (4.2.2) using RStudio (2022.07.02 Build 576).

To further explore the regulation mechanism of melanin synthesis under different light intensities, the transcriptional expression profiles from higher and low light intensities were separately compared. There were two genes (DJ50000770.1 and DJ50011790.1) encoding blue light receptors (white collar: WC) that were highly expressed under high light intensity (Table S4–S6). Some photoreceptor-encoding genes, such as FAD-binding genes (DJ50128910.1, DJ50121620.1, DJ50103310.1, and DJ50058490.1) and the FMN-binding gene nadA (DJ50114790.1), were significantly up-regulated. Moreover, the expression of the photolyase-encoding gene PHR (DJ50074300.1) and probable quinone oxidoreductase-encoding gene ZTA1 (DJ50028000.1) was also up-regulated. In addition, the red-light sensors (phytochromes) genes (DJ50039250.1 and DJ50051180.1) were significantly induced by high light intensity (Figure 9, Table S17). Taken together, these genes associated with light may thus play important roles in melanin synthesis under high light intensity.

### 3.6. Signal Pathways Related to Light

Many signaling pathways are known to play important roles in melanin synthesis. Light signaling can be transmitted to the cell via MAPK signaling. We identified two genes (DJ50133570.1 and DJ50065330.1) involved in MAPK signaling pathways that were significantly up-regulated under high light intensities (Figure 9, Table S17). Moreover, two genes (DJ50104040.1 and DJ50104090.1) involved in the cGMP-dependent protein kinase pathway (cGMP-PKG) showed a trend of increased expression under higher light intensity. These results indicate that high light intensity can significantly activate some signal pathways to promote melanin synthesis.

### 3.7. Genes Involved in Melanin Synthesis Pathways

Melanin synthesis in fungi is a complex process that involves numerous genes. The DEGs identified in the present study were found to be involved in the tyrosine metabolism pathway, which is one of the main melanin synthesis pathways. The tyrosinase-encoding gene *TYR1* (DJ50097310.1, DJ50055220.1, and DJ50097480.1) was significantly up-regulated under higher light intensities. In addition, the expression levels of other genes (DJ50002840.1 and DJ50003220.1) involved in the tyrosine metabolism pathway were significantly increased under higher light intensity (Figure 9, Table S17). The *LAC1* gene (DJ50007430.1 and DJ50035650.1) encoding laccases, which catalyze the oxidation of phenolic substrates, was significantly induced under high light intensity (Figure 9, Table S17). According to these results, we infer that high light intensity can promote melanin synthesis by promoting the expression of genes encoding important enzymes involved in the melanin synthesis pathway.

### 3.8. Validation of RNA-Seq Data by qRT-PCR

**Figure 10:**
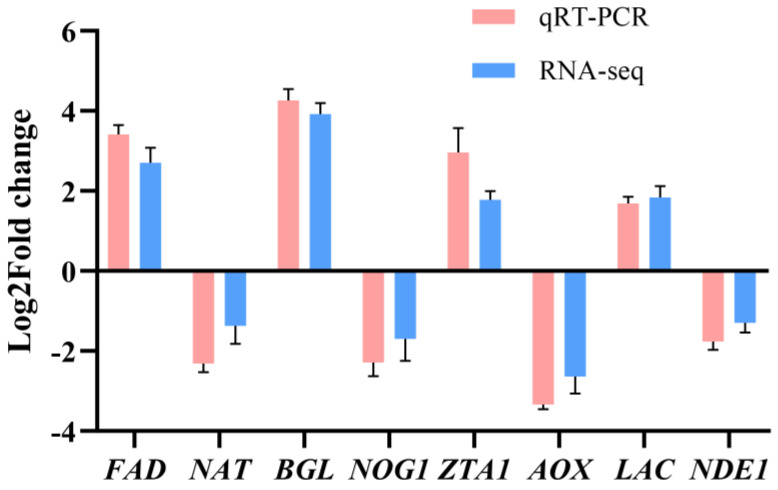
Validation of the DEGs in different comparisons using qRT-PCR. The expression level of each gene was normalized by the level of *GADPH* as reference gene.

qRT-PCR was used to quantitatively validate the relative expression levels of eight DEGs to confirm the gene expression profiles obtained by RNA-seq, including some up-regulated genes and some down-regulated genes identified in the three comparisons. The DEGs exhibited similar expression patterns to those found in the RNA-seq data (B4 vs. B1) (Figure 10). Moreover, the mRNA expression levels of genes related to light, genes in signal pathways related to light, and genes involved in melanin synthesis pathways were confirmed by qRT-PCR (B4 vs. B1). The results showed that these genes would be significantly overexpressed under high light intensity, which was also consistent with RNA seq data (Figure S5). Taken together, these results suggested that the transcriptome data were accurate and reliable.

## 4. Discussion

*A. heimuer* is one of the most productive edible fungi in China. Light is one of the indispensable environmental factors for the development of edible fungi. Studying the influence of light conditions on the pileus color of *A. heimuer* will be of great significance for improving fruiting body traits and its economic value. Many previous works have investigated the regulatory role of light in the biological processes of edible fungi [25,26]. However, there are no specific studies on the relationship between light intensity and the color traits of *A. heimuer*. In the present study, we conducted the first comprehensive transcriptome analysis of *A. heimuer* grown under different light intensities for the purpose of identifying candidate genes involved in melanin synthesis.

A major reason for studying the melanin synthesis of *A. heimuer* is its unique biological activity and high value-added commercial traits. Melanin, a secondary metabolite produced by *A. heimuer*, is usually secreted under restrictive nutritional conditions [27]. However, the higher the degree of black, the higher the commercial value of *A. heimuer*. In our study, we observed that cultivation under high light intensity would significantly increase the blackness of the fruiting bodies of A. heimuer, as the melanin content was higher in them under high light intensity treatment (Figure 1 and Figure 2). Melanin is an active ingredient that protects the fungus from abiotic stresses. Moreover, melanin is an effective photoprotectant that not only absorbs light, but also disperses energy within the structure, thereby protecting the fungus from intense light damage [28]. Therefore, large amounts of melanin under high light intensity play a role in resisting light stress. Based on these observations, we speculate that a high content of melanin contributes to the adaptability of *A. heimuer* to a high light intensity environment.

To better adapt to environmental changes, fungi have evolved a sophisticated light perception system, including the presence of light, light direction, light intensity, light wavelengths, and light period [29]. The main output form of the light response depends on the control of the expression of genes at the cellular level. The changes in melanin content resulting from the photoresponse must involve the activation of photoreceptors, light signal transduction, and melanin synthesis. First, the transmission of light signals and activation of downstream pathways are dependent on many photoreceptors and associated proteins [30]. Photoreceptors are ubiquitous in filamentous fungi, such as flavin-binding blue light receptors, retinal-containing green light sensors, and red light sensors (phytochromes) with a linear tetrapyrrole [31,32]. The blue-light receptor WC-1 contains specific flavin-binding motifs, which are widely distributed in filamentous fungi, that work in cooperation with the WC-2 protein, forming a complex and jointly participating in activation of the downstream melanin synthesis pathway [33]. In this study, we found that high light intensity conditions can activate the expression of these two photoreceptor genes and FMM- and FAD-binding genes in *A. heimuer*, which provides the basis for a large amount of intracellular melanin synthesis. Thus, it is possible that with the increase in light intensity, the light wavelength that can promote the synthesis of melanin by fungi also increases, thereby further activating the expression of photoreceptors related to melanin synthesis pathways. Moreover, our study showed that high light intensity will activate many other light receptor proteins, thus activating more metabolic pathways to help *A. heimuer* better adapt to a strong light environment.

Through the conversion of photoreceptors, fungi can adapt to a changing light environment by converting the light signal into a chemical signal. Light signaling transduction mainly occurs through the MAPK pathway. MAPK signal transduction pathways are ubiquitous in eukaryotes and are evolutionarily conserved, playing important roles in the light signal-mediated regulation of melanin synthesis [34]. We identified various genes involved in the MAPK signaling pathway to be significantly up-regulated under high light intensities. Therefore, high expression of MAPK signal pathway genes in *A. heimuer* under high light intensity will lead to activation of downstream melanin synthesis pathways. In particular, the production of tyrosinase in the melanin synthesis pathway can be regulated by the cGMP-PKG signaling pathway, thus promoting the synthesis of melanin [35]. A previous study also found that WC-dependent blue light signaling was connected to stress signaling, such as the high-osmolarity glycerol response (HOG) pathway [36]. Melanin synthesis is closely related to the HOG pathways [37]. Therefore, the activated photoreceptors under high light intensity will connect with signaling cascades and directly lead to the deposition of mycelial melanin to ultimately deepen the color of *A. heimuer*. In addition, the response of other signaling or metabolic pathways under high light intensity can also help *A. heimuer* better adapt to the environment and grow normally. For instance, activation of the AMPK signaling pathway is useful for glucose uptake and utilization [38], and the peroxisome pathway is important in resisting oxidative stress.

Tyrosinase is an oxidase that acts as a rate-limiting enzyme for melanin synthesis [39]. Laccase, as a copper-containing oxidase, has many functions, including the polymerization/degradation of lignin, the pathogenicity of fungi, pigmentation, and melanin formation [40]. Laccases are known to be involved in the biosynthesis of both DHN-melanin and DOPA melanin [41]. In the present study, laccase-encoding genes and tyrosinase-encoding genes, such as *LAC1*, *TYR1,* and *MELC2,* in the fruiting bodies were significantly induced by high light intensities. These two enzymes are involved in the catalytic reaction of the melanin synthesis pathway of many fungi [42]. Therefore, the high expression of these two enzymes under high light intensity will directly lead to an increase in melanin synthesis, resulting in a darker color of *A. heimuer*.

## 5. Conclusions

Here, we provide the first report of the effect of light intensity on the fruiting body color of *A. heimuer* and identify candidate genes involved in light intensity-induced melanin synthesis. In summary, the present study provides new insight into the mechanism by which light intensity regulates the melanin synthesis of *A. heimuer* based on transcriptomics technology. The formation of a blacker fruiting body is dependent on light sensing, signal transduction, and melanin synthesis processes. We speculate that the significantly up-regulated DEGs enriched in these processes may be related to melanin synthesis, and the regulatory mechanism of light intensity on melanin synthesis appears to be highly complex. These findings can thus inform additional studies to further our understanding of how *A. heimuer* regulates melanin synthesis under high light intensity, establishing a research foundation for elucidating the complex regulatory mechanism of light signals. Moreover, the present findings have important implications for genetic breeding or light environmental regulation of other black-colored edible fungi.

## Figures and Tables

**Table 1:** Color reading of the fruiting bodies collected from different light intensity treatments.

Samples	L*	a*	b*
B1	31.03 ^a^	6.06 ^a^	7.26 ^a^
B2	25.7 ^b^	1.6 ^b^	6.73 ^a^
B3	22.3 ^c^	3.33 ^ab^	4.8 ^a^
B4	19.6 ^d^	5.03 ^ab^	4.33 ^a^

Note: Numeric description of color using the L*a*b* CIELAB color space. L* (lightness or darkness) values range from black (0) to white (100); a* represents the color direction in red (a* > 0) or green (a* < 0); and b* represents the color direction in yellow (b* > 0) or blue (b* < 0). Values with different superscript uppercase letters within the same column are significantly different (*p* ≤ 0.05).

## Data Availability

All data presented in this study are available.

## Supplementary Materials

The following supporting information can be downloaded at: https://www.mdpi.com/article/10.3390/cells12010056/s1, Figure S1: Boxplot of FPKM distribution in samples; Figure S2: The heatmap of significant DEGs under different light intensities; Figure S3: Venn diagram of the DEGs among three comparisons (B2 vs. B1, B3 vs. B1, B4 vs. B1). B2_B1 means B2 vs. B1, B3_B1 means B3 vs. B1, and B4_B1 means B4 vs. B1; Figure S4: Number of KEGG pathways enriched for three comparisons; Table S1: The list of primers for qRT-PCR validation; Table S2: Summary of raw sequencing data and assembly; Table S3: Mapped results of clean sequencing data; Table S4: Clustering analysis of significant DEGs in the B2 vs. B1 comparison; Table S5: Clustering analysis of significant DEGs in the B3 vs. B1 comparison; Table S6: Clustering analysis of significant DEGs in the B4 vs. B1 comparison; Table S7: The common or unique DEGs of the three comparisons; Table S8: GO enrichment analysis of the DEGs in the B2 vs. B1 comparison; Table S9: GO enrichment analysis of the DEGs in the B3 vs. B1 comparison; Table S10: GO enrichment analysis of the DEGs in the B4 vs. B1 comparison; Table S11: The functional categories of the KEGG pathway enriched for the B2 vs. B1 comparison; Table S12: The functional categories of the KEGG pathway enriched for the B3 vs. B1 comparison; Table S13: The functional categories of the KEGG pathway enriched for the B4 vs. B1 comparison; Table S14: KEGG pathways of the significantly up-regulated DEGs in B2 vs. B1; Table S15: KEGG pathways of the significantly up-regulated DEGs in B3 vs. B1; Table S16: KEGG pathways of the significantly up-regulated DEGs in B4 vs. B1; Table 17: Annotation information of possible genes related to light intensity and melanin synthesis.
