# Supplementary material for: Mechanism Underlying Light Intensity-Induced Melanin Synthesis of Auricularia heimuer Revealed by Transcriptome Analysis
Source: Cells. 2022 Dec 23;12(1):56. doi: 10.3390/cells12010056 (PMC9818193; doi:10.3390/cells12010056)
Supplement: Supplementary file 1 [file cells-12-00056-s001.zip › Supplementary file.pdf]

Mechanism underlying light intensity-induced melanin synthesis of *Auricularia heimuer* revealed by transcriptome analysis

Zhiheng Qiu [1,2,†], Yanliang Gao [1,2,†], Shuang Wang [1,2], Jun Wang [1,2], Xinyi Wang [1,2], Nuo Cai [1,2], Jiazhi Zhao [1,2], Tingshu Li [1,2], Hongpeng Li [1,2], Tianlai Li [1,2] and Lili Shu [1,2*]

1 College of Horticulture, Shenyang Agricultural University, Shenyang, 110866, PR China; qiuzh@syau.edu.cn; 2020240423@stu.syau.edu.cn; 2021240482@stu.syau.edu.cn; 2020240419@stu.syau.edu.cn; 2021220395@stu.syau.edu.cn; 2021240414@stu.syau.edu.cn; jiazhi@stu.syau.edu.cn; 2021240473@stu.syau.edu.cn; 2022240532@stu.syau.edu.cn; tianlaili@126.com

2 Key Laboratory of Protected Horticulture of Education Ministry and Liaoning Province, Shenyang, 110866, PR China.

* Correspondence: shulili1986@syau.edu.cn (L.S.); Tel.: +86-024-88487143 (L.S.)

† These authors contribute equally to this work.

Figure S1. Boxplot of FPKM distribution in samples.

Figure S2. Venn diagram of the DEGs among three comparison (B2 vs. B1, B3 vs. B1, B4 vs. B1). B2_B1 means B2 VS B1, B3_B1 means B3 VS B1, and B4_B1 means B4 VS B1.

Figure S3. The heatmap of significantly DEGs under different light intensities.

Figure S4. Number of KEGG pathways enrichment for three comparison.

Table S1. The list primers for qRT-PCR validation.

Gene ID	Description	Primers sequence (5'-3')
DJ50080610.1	Glyceraldehyde 3-phosphate dehydrogenase (*GAPDH*)	Forward: GCATCGGGCGGATTGTGA<br>Reverse: GCTTGCCGTCCTTGGTCT
DJ50128910.1	FAD binding domin (*FAD*)	Forward: CAGCCCTTAGCGAGGTGAAA<br>Reverse: CTTCCTGGACTGTCTAGCCG
DJ50019860.1	Enoyl-CoA hydratase/isomerase (*NOG1*)	Forward: AGCTCAAGGAGCTCACGTTC<br>Reverse: GCACATCATCGCTAGTTCGC
DJ50083280.1	Glucan 1,3-beta-glucosidase (*BGL*)	Forward: AGTTGTACTGCACGAGGACG<br>Reverse: TCAACCAGAATGCGAACCGA
DJ50039520.1	Acyl-CoA N-acyltransferase (*NAT*)	Forward: CTCGTACTCCTCGACGAAGC<br>Reverse: CTACAACGAGCCGCTCTTCA
DJ50028000.1	Quinone oxidoreductase (*ZTA1*)	Forward:

		GGGCACATACGACTCGTGAA<br>Reverse:<br>GGAAAGGACACTACAGCCCC
DJ50033930.1	Alcohol oxidase (*AOX*)	Forward:<br>TCGAGTGGTGCCATTCAGTC<br>Reverse:<br>TGTCCCAGGTGACGATTGTG
DJ50007430.1	Laccase (*LAC*)	Forward:<br>AGAACGCAAAAACTGCCGAC<br>Reverse:<br>TGTCGAACAGGACGTTGAGG
DJ50116550.1	Pyridine nucleotide-disulphide oxidoreductase (*NDE*)	Forward:<br>GTTCCTCAAGACGCTCGACA<br>Reverse:<br>GACCATAACCTGGCGCTTCT
DJ50121620.1	putative flavohemoglobin	Forward:<br>TCCTGACGCTACGACGACTC<br>Reverse:
DJ50103310.1	FAD/NAD(P)-binding domain-containing protein	Forward:<br>ATCGGCGTACTTGCTGTTCA<br>Reverse:<br>CGTCCTCGTGCAGTACAACT
DJ50058490.1	FAD/NAD(P)-binding domain-containing protein	Forward:<br>TGGAGATGGCGTTGCGATTA<br>Reverse:<br>TTACCGGATGCTGACCCCTA
DJ50114790.1	FMN-linked oxidoreductase	Forward:<br>TAGGGGTCAGCATCCGGTAA<br>Reverse:<br>TCATGGGCGACATGGTCATC
DJ50074300.1	Photolyase	Forward:<br>TACAATCCCGCGCCGTAAAT<br>Reverse:<br>TGAACAGCAAGTACGCCGAT
DJ50039250.1	Domain present in phytochromes and cGMP-specific phosphodiesterases	Forward:<br>GACTCGGACTGTATGACGCC<br>Reverse:<br>GTCGCACTGAGACATCGACA
DJ50051180.1	Phytochrome region	Forward:<br>AACGTTGTCGAGCACAAAGC<br>Reverse:<br>GGCCCGTGTGGCTATTCTAT
DJ50133570.1	Putative serine/threonine phosphatase	Forward:<br>CGTAGTTCTCGTACTGCGGG

	Reverse: TCTATTCCGGCAGCAACTCG
DJ50065330.1 Putative serine/threonine phosphatase	Forward: ATACAATCCCGCGCCGTAAA<br>Reverse: GTCATACAGTCCGAGTCGCC
DJ50104040.1 ADP,ATP carrier protein	Forward: GTAAGGTCATGCTCGCTGGT<br>Reverse: AAACTCGAGCCATTCGTCGT
DJ50104090.1 ADP,ATP carrier protein	Forward: AACGAGGCTCTCCGAAGTTG<br>Reverse: GCACCTTTCTGCCGTTTAGC
DJ50097310.1 Tyrosinase central domain protein	Forward: CGCCGCTCTCAGTCTTACTT<br>Reverse: AACTTCGGAGAGCCTCGTTG
DJ50055220.1 Tyrosinase	Forward: AGGCCAGCCTGGAAGTTTAC<br>Reverse: TGTTCAGGTCGATAGCAGGC
DJ50097480.1 Tyrosinase	Forward: GCTGGTGCTGTCGAGTATGA<br>Reverse: GAACCATTGCGAGAACGACG
DJ50002840.1 Succinate-semialdehyde dehydrogenase	Forward: CAATGGTTCTCGGAGGCGAT<br>Reverse: TATCACGGTTCGAGTTGGGC
DJ50003220.1 Succinate-semialdehyde dehydrogenase	Forward: CGAGCTACGTTCAGGTCGTT<br>Reverse: CAGCGAGCATGACCTTACGA

Table S2. Summary of raw sequencing data and assembly.

Sample	Reads length (bp)	Raw reads	Raw data (G)	Q20 (%)	Q30 (%)	GC (%)
B1.1	150	54,091,688	8.11	96.85	92.30	59.22
B1.2	150	53,419,152	8.01	96.99	92.47	59.44
B1.3	150	51,969,936	7.80	96.95	92.51	59.19
B2.1	150	53,164,826	7.98	97.04	92.80	59.11
B2.2	150	47,502,904	7.13	96.96	92.57	59.21

B2.3	150	52,211,954	7.83	96.88	92.32	59.48
B3.1	150	43,538,158	6.53	97.11	92.92	59.54
B3.2	150	48,300,946	7.25	96.72	91.96	59.55
B3.3	150	46,903,940	7.04	96.78	92.34	59.35
B4.1	150	53,379,006	8.01	96.87	92.29	59.56
B4.2	150	54,033,966	8.11	96.87	92.34	59.31
B4.3	150	42,381,642	6.36	96.90	92.44	59.68

Table S3. Mapped results of clean sequencing data.

Sample	Total reads after filtered	Mapped on reference		Multiple mapped		Uniquely Mapped	
		**Reads**	**%**	**Reads**	**%**	**Reads**	**%**
B1.1	48,260,332	40,059,100	83.01	2,266,715	4.70	34,487,854	64.55
B1.2	47,523,218	39,400,816	82.91	1,274,969	2.68	35,356,380	66.80
B1.3	43,763,916	35,940,342	82.12	1,178,787	2.69	34,153,786	66.23
B2.1	46,152,856	38,784,733	84.04	1,684,189	3.65	35,596,760	67.60
B2.2	41,639,170	34,922,221	83.87	1,486,036	3.57	31,682,853	67.43
B2.3	45,463,188	37,937,965	83.45	1,312,710	2.89	34,803,707	67.39
B3.1	38,984,316	32,925,696	84.46	1,030,054	2.64	29,750,448	69.98
B3.2	42,579,128	35,448,356	83.25	1,005,385	2.36	32,250,959	67.45
B3.3	39,028,800	32,749,602	83.91	1,240,031	3.18	31,517,418	68.24
B4.1	45,959,064	38,718,421	84.25	1,355,596	2.95	35,863,496	67.96
B4.2	46,110,028	38,712,272	83.96	1,241,494	2.69	36,517,004	68.42
B4.3	37,788,408	31,850,161	84.29	1,207,431	3.20	2,266,283	67.65
