# Supplementary figures and images for: Mechanism Underlying Light Intensity-Induced Melanin Synthesis of Auricularia heimuer Revealed by Transcriptome Analysis
Source: Cells. 2022 Dec 23;12(1):56. doi: 10.3390/cells12010056 (PMC9818193; doi:10.3390/cells12010056)

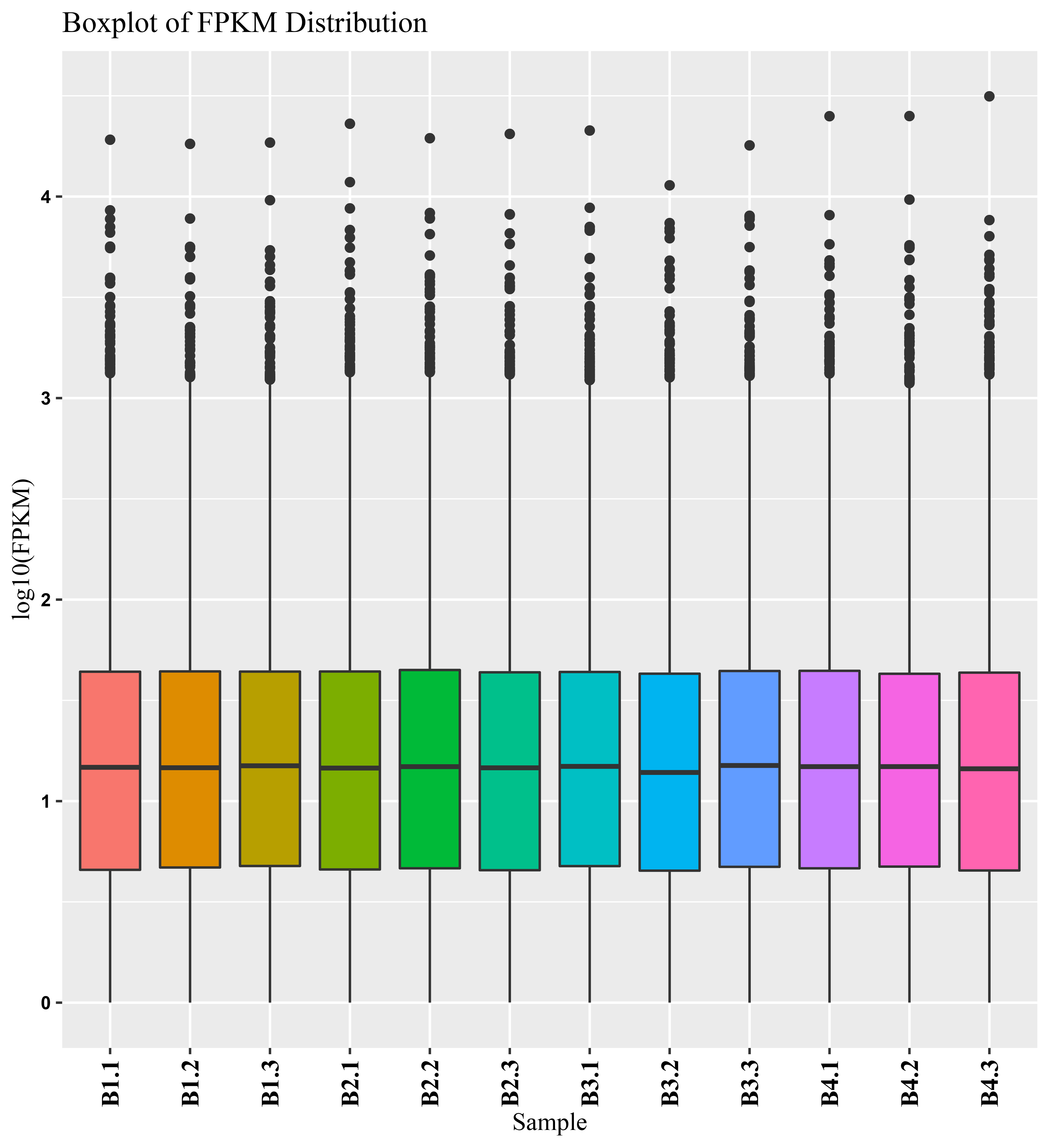

Supplement: Supplementary file 1 [file cells-12-00056-s001.zip › Figure S1.tif]

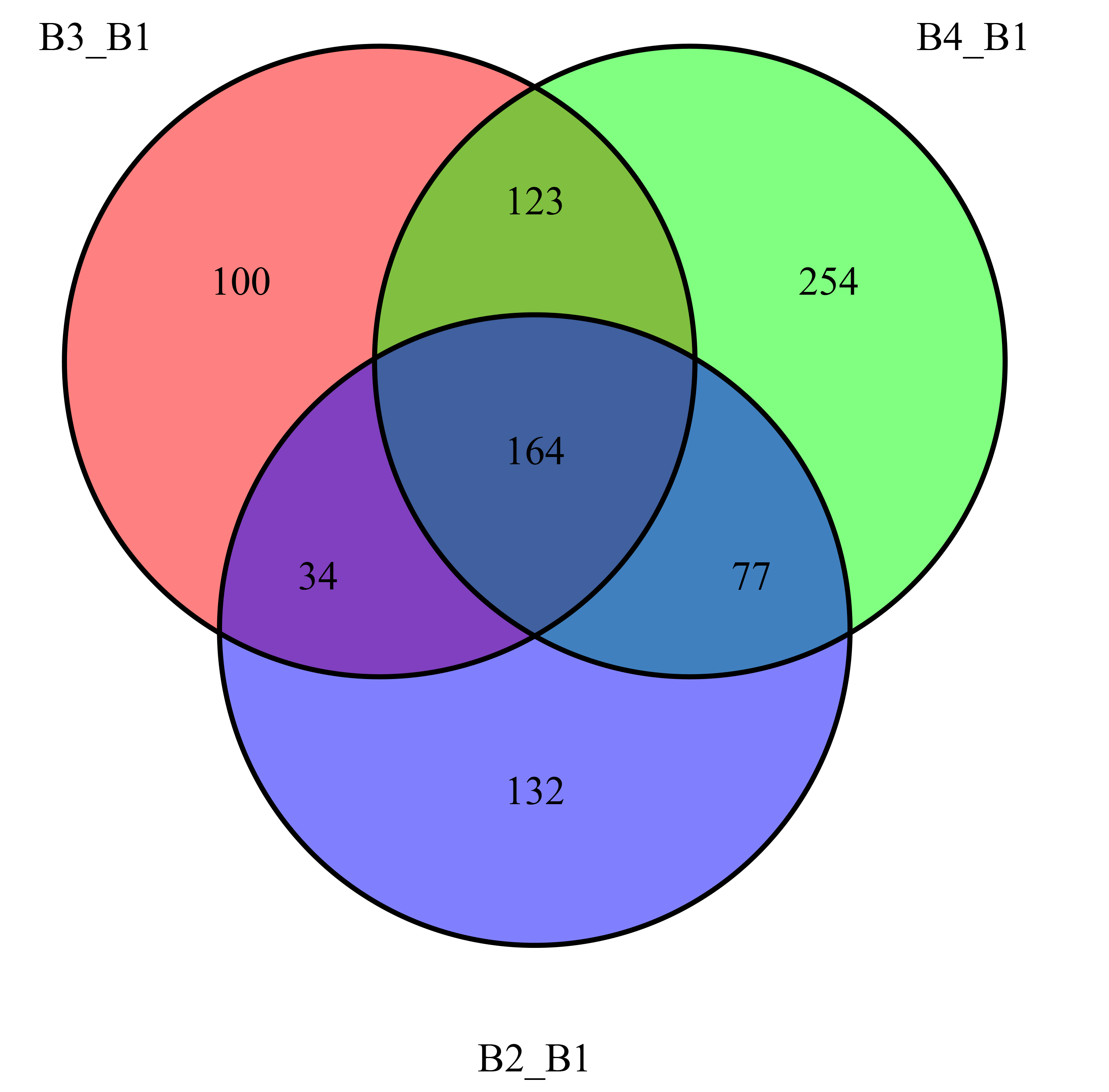

Supplement: Supplementary file 1 [file cells-12-00056-s001.zip › Figure S2.tif]

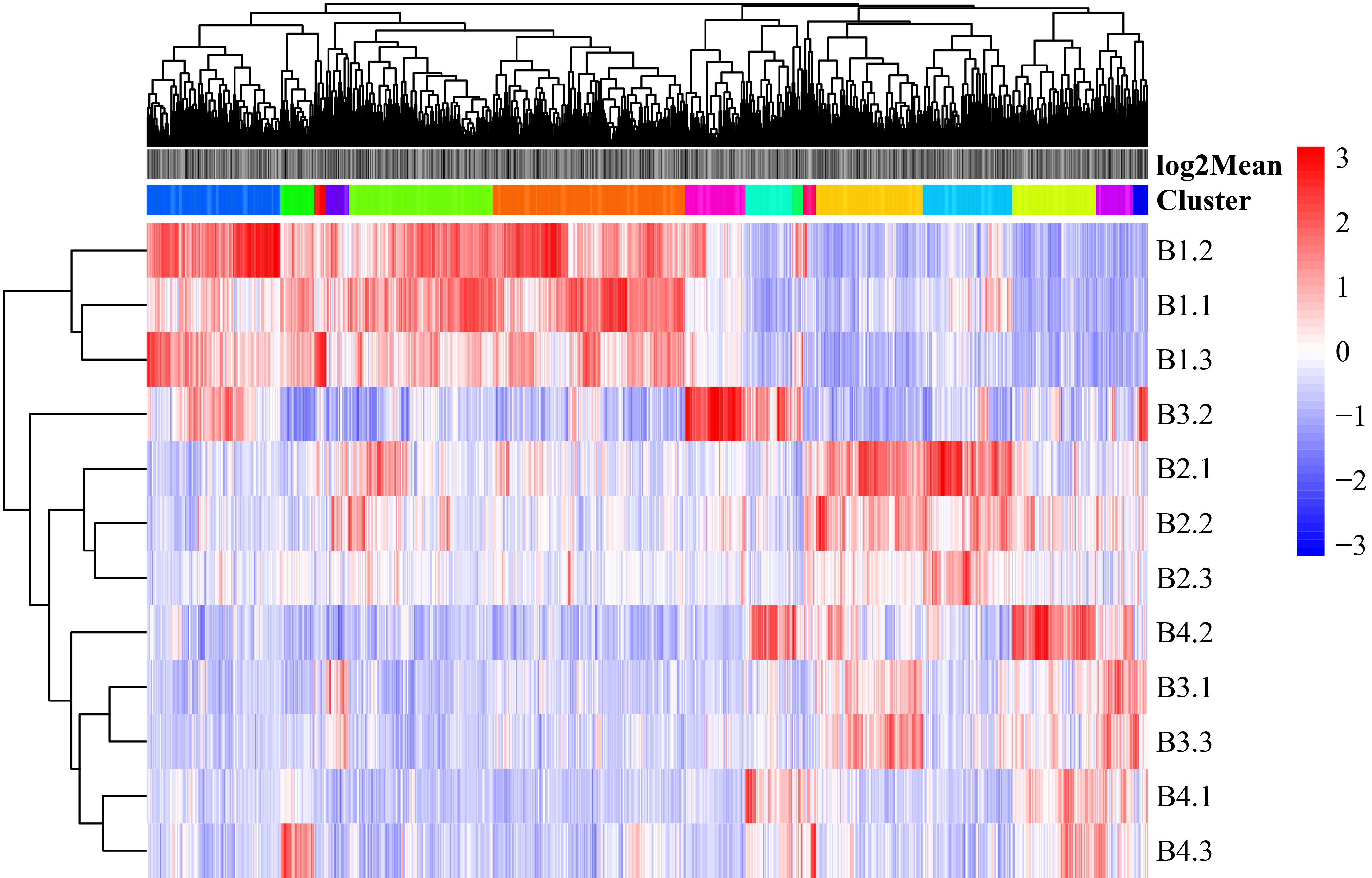

Supplement: Supplementary file 1 [file cells-12-00056-s001.zip › Figure S3.tif]

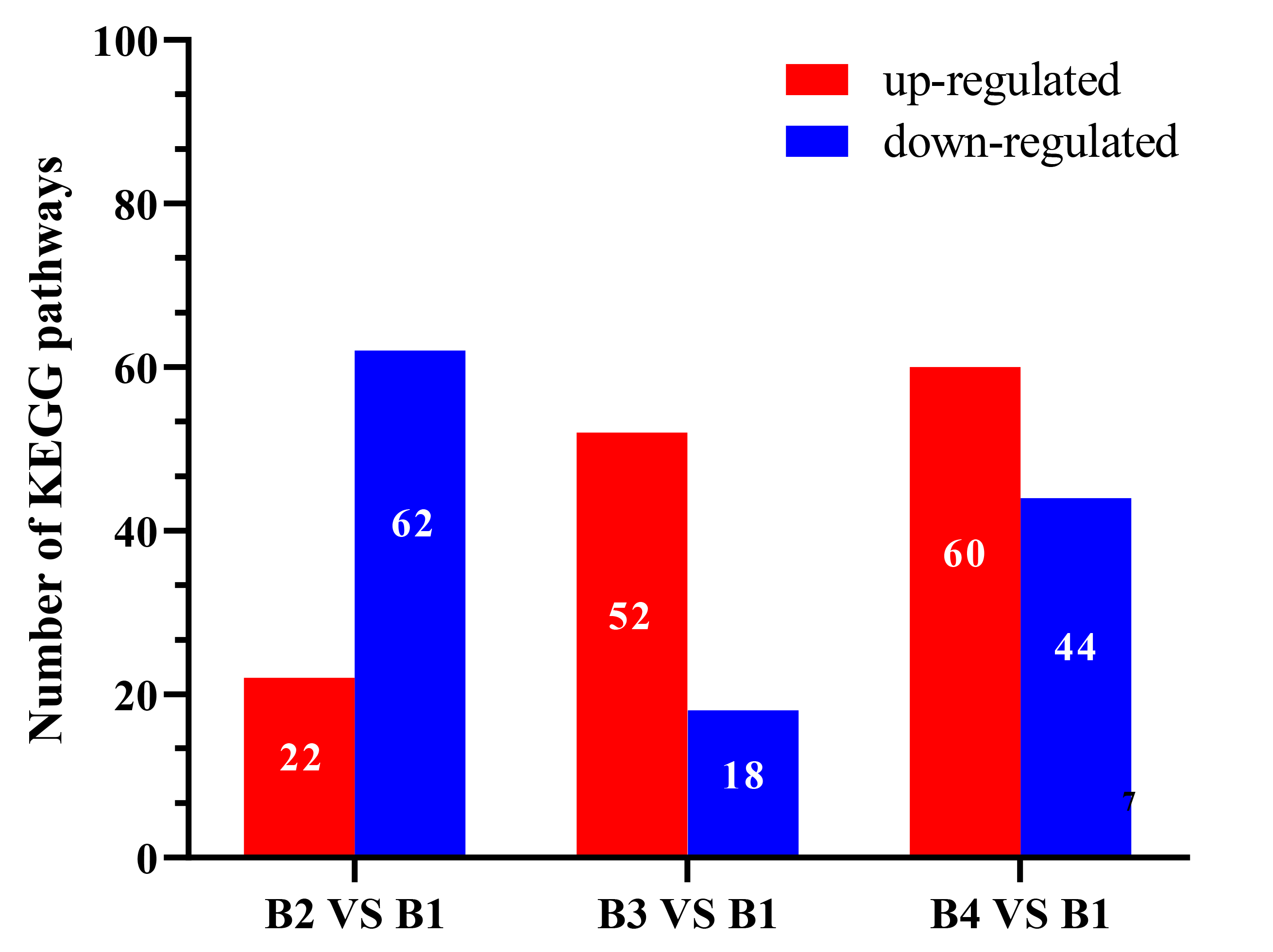

Supplement: Supplementary file 1 [file cells-12-00056-s001.zip › Figure S4.tif]

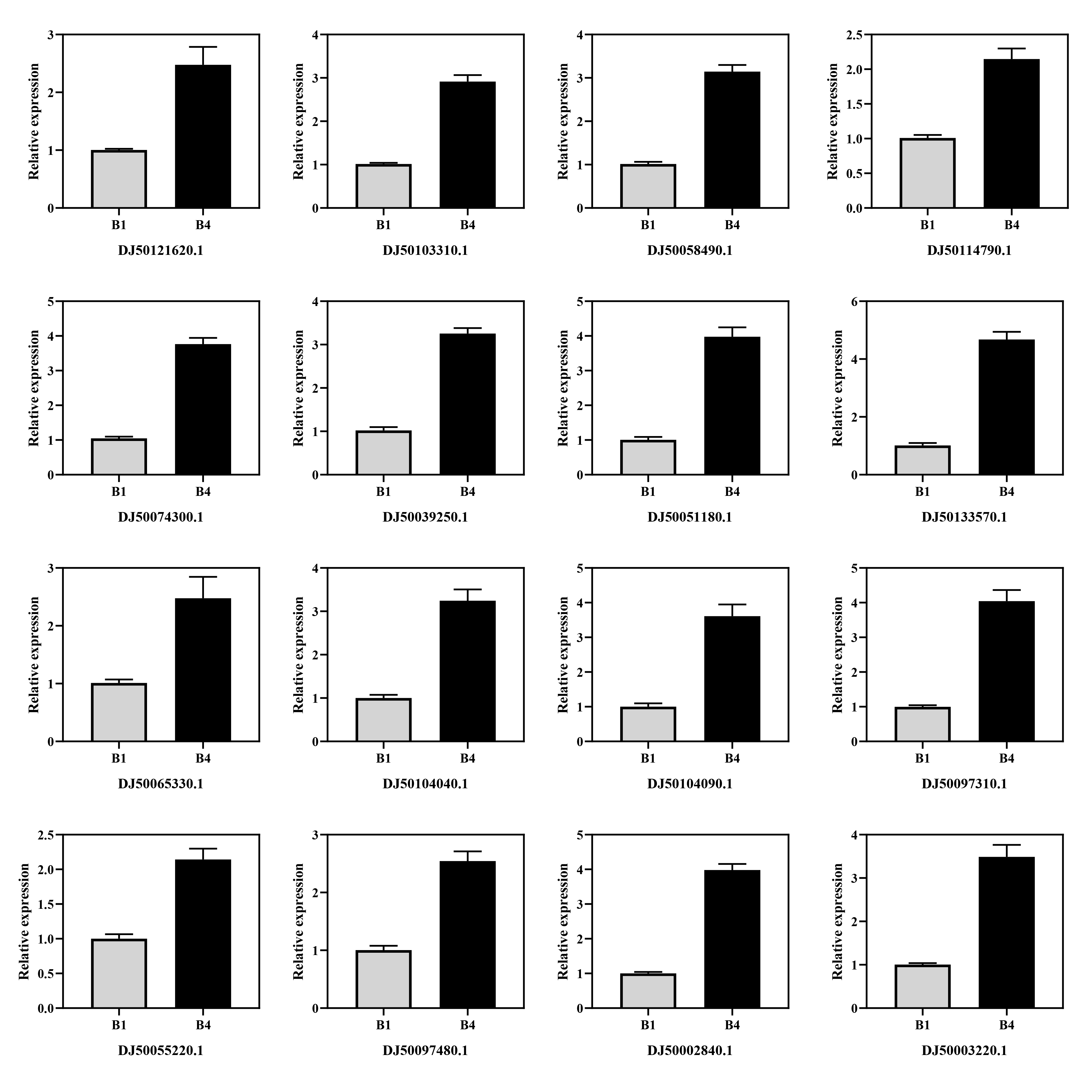

Supplement: Supplementary file 1 [file cells-12-00056-s001.zip › Figure S5.tif]
